# Supplementary material for: Long-read RNA sequencing identifies region- and sex-specific C57BL/6J mouse brain mRNA isoform expression and usage
Source: Mol Brain. 2024 Jun 20;17:40. doi: 10.1186/s13041-024-01112-7 (PMC11188239; doi:10.1186/s13041-024-01112-7)

Supplementary Figure 1. Novel transcripts are expressed more highly than annotated transcripts. Density plot showing expression levels (measured in counts per million, CPM) between the novel (yellow) and annotated (blue) transcripts. Density is calculated by kernel density estimation. When we ran a one-sided Wilcoxon rank sum test at 5 mean CPM thresholds (**A** - 0 CPM, **B** - 1 CPM, **C** - 2 CPM, **D** - 5 CPM, and **E** - 8 CPM), the two distributions were significantly different at every level.

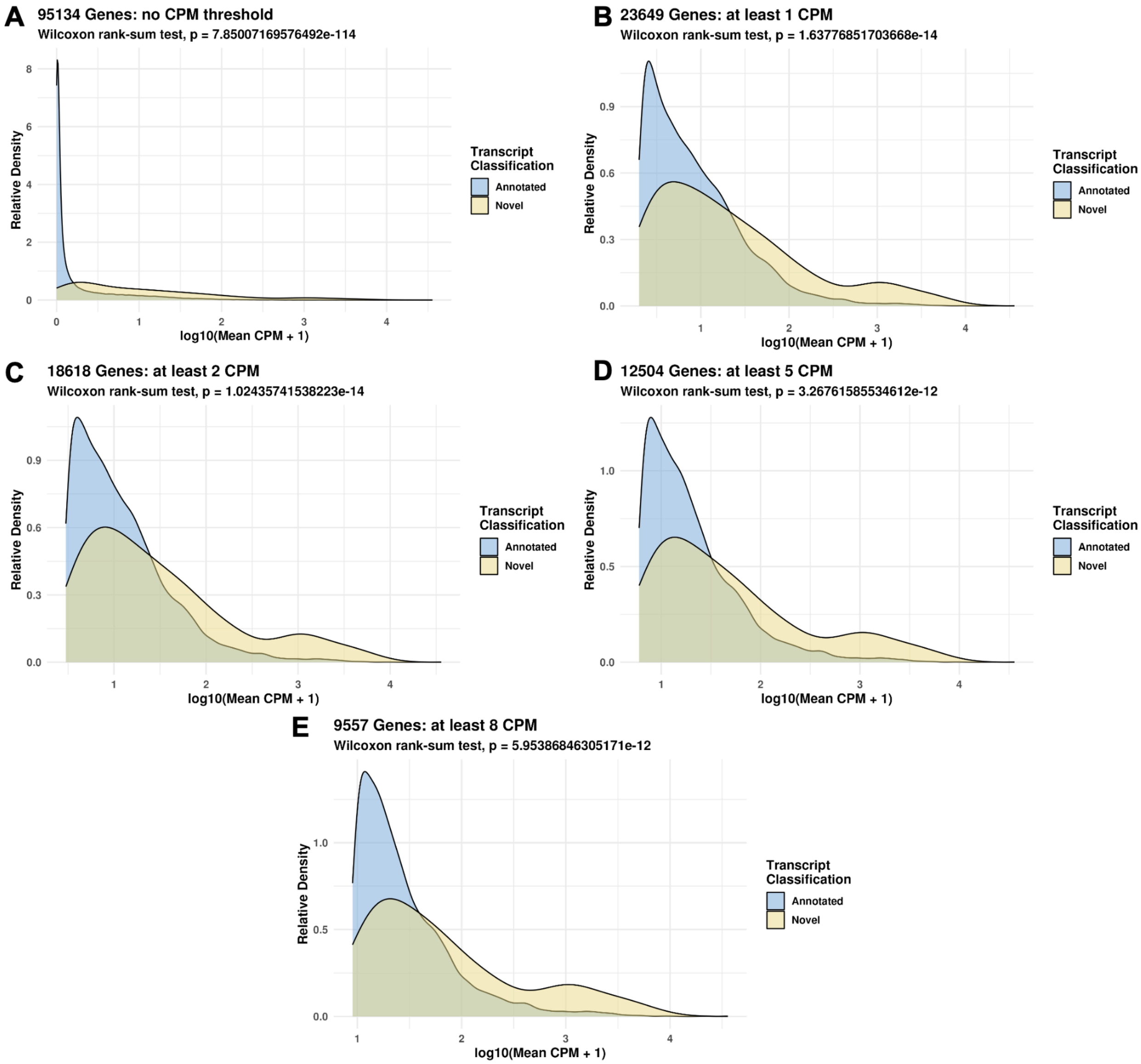

Supplementary Figure 2. Genes with DTU across sex for the whole brain. SwitchPlots showing transcript summary, gene expression, isoform expression, and isoform usage of the genes for all brain regions across males (M; dark gray) and females (F; light gray). Genes shown are *Zfp324* (**A**), *Zfp862-ps* (**B**), *Shisa5* (**C**), and unannotated gene or *Gm10605* (**D**).

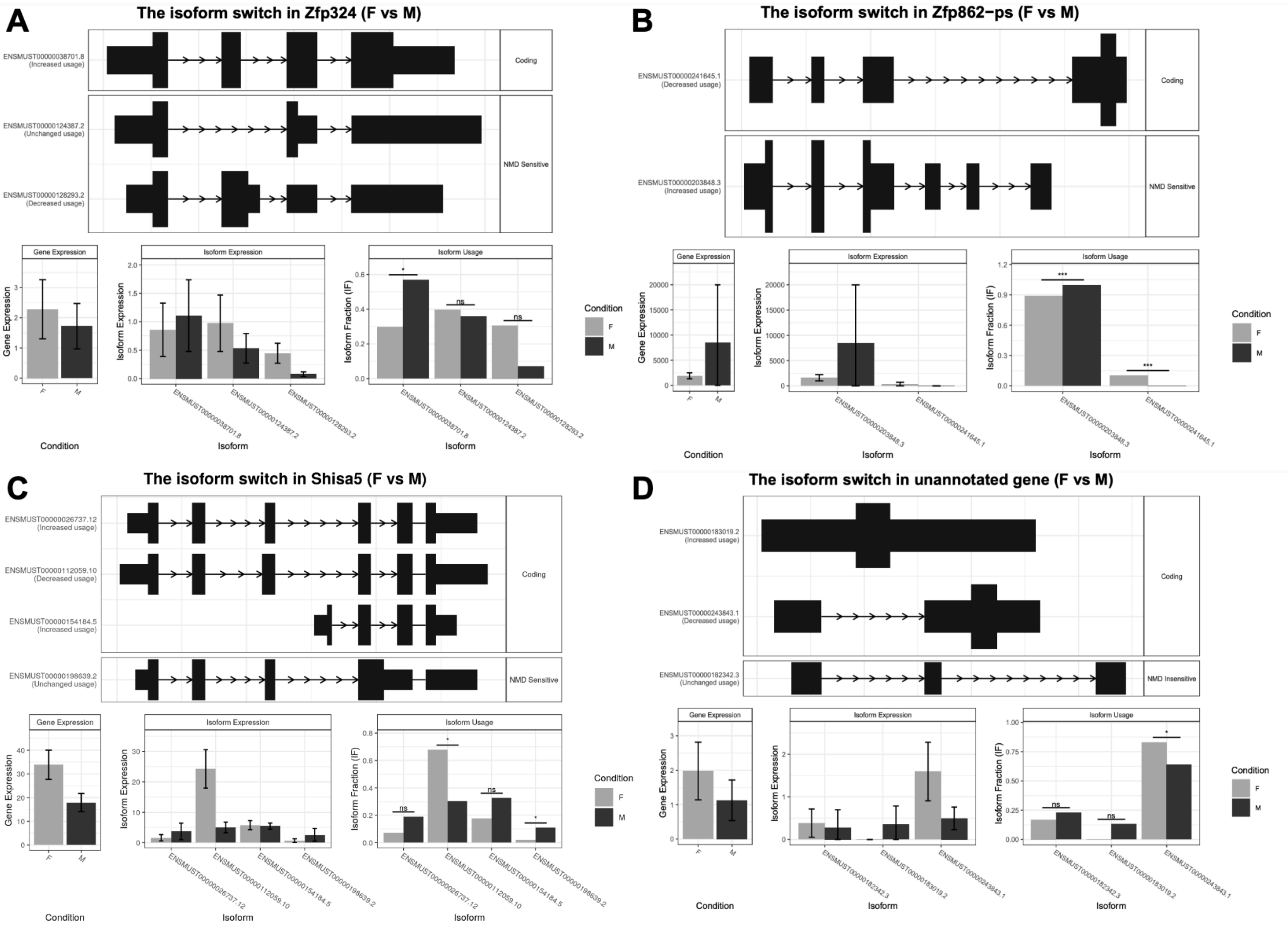

Supplementary Figure 3. *Tsr2* has a non-significant presence or absence of female-specific transcripts in the hippocampus. SwitchPlot showing transcript summary, gene expression, isoform expression, and isoform usage of the genes for all brain regions across males (M; dark gray) and females (F; light gray).

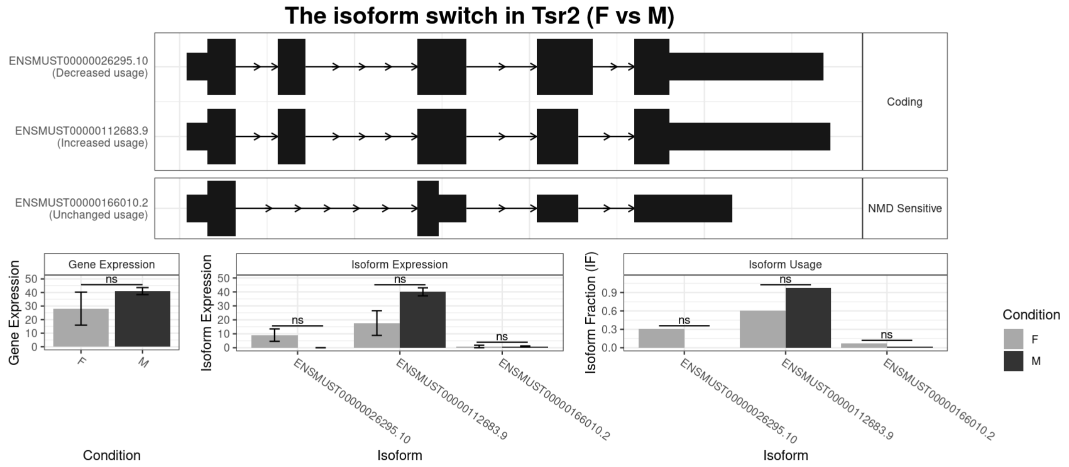

Supplement: Supplementary file 8 — Additional file 8. Supplementary Figures. This PDF contains supplementary figures and their figure legends. [file 13041_2024_1112_MOESM8_ESM.pdf]
